# Long-Term Violent Reoffending Following Forensic Psychiatric Treatment: Comparing Forensic Psychiatric Examinees and General Offender Controls

**DOI:** 10.3389/fpsyt.2019.00715

**Published:** 2019-10-16

**Authors:** Susanne Bengtson, Jens Lund, Michael Ibsen, Niklas Långström

**Affiliations:** ^1^Department of Forensic Psychiatry, Aarhus University Hospital Psychiatry, Aarhus, Denmark; ^2^Sexological Clinic, Psychiatric Centre Copenhagen, Copenhagen, Denmark; ^3^I2 Minds, Aarhus, Denmark; ^4^Department of Medical Epidemiology and Biostatistics, Karolinska Institutet, Stockholm, Sweden

**Keywords:** forensic psychiatric patients, violent reoffending risk, facets of violence, long-term follow-up, forensic psychiatric evaluation

## Abstract

**Background:** Long-term violent re-offending in forensic psychiatric (FP) patients *vs*. non-FP offenders is largely unknown.

**Methods:** We studied rates and facets of long-term violent reoffending among 1,062 violent forensic psychiatric examinees (FPE) consecutively undergoing pre-trial, forensic psychiatric examination (FPE) in Denmark during 1980–1992. Altogether, 392 were sentenced to *FP treatment* (FPE+T); the remaining 670 examinees received ordinary non-FP sanctions (FPE-T). FPE+T were compared to 392 contemporary *matched violent general offenders* (GEN) without FPE or other psychiatric contacts and sentenced to ordinary non-FP sanctions. FPE data were linked to population-based registers with sociodemographic, psychiatric, and crime information, and we estimated relative risks controlling for birth year, sex, educational and marital status, and previous violent crime.

**Results:** During follow-up (mean = 18.0–19.5 years), FPE+T and GEN had *any violent recidivism* rates of 43% *vs*. 29% [adjusted hazard ratio (aHR) = 1.5; 95% CI, 1.1–1.9], respectively. Corresponding findings for *severe violence* (21% *vs*. 14%; aHR = 1.3; 95% CI, 0.9–1.9) and *recurrent violence* (3+ violent convictions; 16% *vs*. 6%; adjusted odds ratio [aOR] = 2.5; 95% CI, 1.5–4.4) also suggested weakly to moderately increased risks in FPE+T, albeit non-significantly for the former. Comparing FPE+T to FPE-T suggested *decreased* risk of *any violence* (43% *vs*. 51%; aHR = 0.8; 95% CI, 0.6–1.1), *severe* (21% *vs*. 34%; aHR = 0.6; 95% CI, 0.4–0.8), and *recurrent violence* [16% *vs*. 22%; adjusted odds ratio (aOR) = 0.7; 95% CI, 0.5–1.0] in FP patients, though non-significantly for any violence and recurrent violence. Among all FPE examinees, violent reoffending was independently predicted by male sex, younger age, pre-index violent crime, personality disorder (*vs*. schizophrenia spectrum and other psychiatric disorder), substance use disorder, and 5+ hospital admissions.

**Conclusion:** FPE examinees, untreated followed by treated, reoffend violently more often than GENs. Similar trends are suggested also for severe and recurrent violence suggesting a need for continua of services for FPE examinees, independently of medico-legal status (i.e., sentencing to treatment or not).

## Introduction

Due to mental ill health, a small but important number of criminal offenders are committed to court-ordered psychiatric treatment. Recently, many Western countries including Denmark have experienced substantial increases in the number of forensic psychiatric (FP) patients (1–4). However, throughout Europe (5) and North America, FP patient care is organized and administered differently due to dissimilar legislation, judicial and court practices, and organization of forensic and mental health services. Such variability even occurs across otherwise quite comparable Nordic welfare states (6). In clinical practice with FP patients, on the other hand, treatment goals are generally two-sided—optimal treatment of psychopathology and reduction of criminal re-offending (7). However, complex comorbidity with neurodevelopmental conditions and substance misuse, socio-economic disadvantage, trauma and neglect, and fluctuating compliance with interventions complicates psychiatric care and risk management and increases reoffending risk (5, 8–10). These aspects also affect mental health budgets disproportionately, despite FP patients being a minority among psychiatric patients (11).

Prior research investigated criminal reoffending in FP patients (12–17). Naturally, reoffending figures increase with extended follow-up to reconviction rates of 40–50% in any crime (12, 18) and violent reoffending alike (12, 14, 16). Prevention of violence recurrence includes also qualitative aspects such as of imminence, severity, and recurrence of violent reoffending and concerns regulatory authorities, service providers, and clinicians alike. However, Douglas and Ogloff (7) reported lower reliability and incremental validity of clinicians’ HCR-20-based (19) structured professional judgments of imminence and severity of violent recidivism among 100 FP patients following discharge, compared to the assessment of any violent reoffending risk. Beyond insufficient rater training and risk communication structures, poor knowledge about base rates of these violence facets might be critical. Monahan (20) argued that proper base rate information of a predicted outcome might be the most important single piece of information needed for risk assessments. However, humans tend to ignore generic and general base rate data and instead focus case-specific information, referred to as *base rate neglect* (21). Similarly, Coid and co-workers (22) reminded that studies of FP patients require sufficient sample size and statistical power to quantify long-term reoffending risks following psychiatric treatment and identify those at highest risk and, specifically, address violent re-offending in FP patients with one or more violent offences at baseline. Beyond a few exceptions (22–25), studies of violent offenders rarely investigated violence risk as multifaceted constructs, including also information on the imminence, severity, and recurrence of violent reoffending, although these aspects might be particularly helpful to forensic psychiatry clinicians (7).

Another long-standing question is whether offenders with severe or major mental illness, usually defined as schizophrenia spectrum or bipolar disorders, are at higher reoffending risk than those without. A recent systematic review of 35 studies following patients discharged from secure hospitals (n=12,056), of which 53% were violent offenders found *overall reoffending rates* of 0–24,244 per 100,000 patient years, a pooled mean estimate of 4,484 per 100,000 patient years and substantial heterogeneity across studies (11). Reported *violent reoffending* rates ranged from 273 to 8,403 per 100,000 person years with a pooled mean of 3,902 and showed considerable heterogeneity across studies (*k* = 15). The findings suggested that FP patients have *lower* reoffending rates overall than comparison subjects such as released prisoners. However, comparisons are difficult as FP patients constitute a highly selected sample who often commit more severe or violent, but not necessarily more frequent, offences and are incapacitated for longer periods at baseline than non-FP offenders. They often have supportive treatment or aftercare, usually associated with lower reoffending risk. Further, between-country comparisons are difficult because of different legislation, recording, reporting and sentencing practices, heterogeneous samples with both prisoners and probationers, varying follow-up periods, and outcome definitions (12, 26). Fazel et al. (11) attempted to adjust for some of these confounders by matching violent FP patients with prisoners having received longer sentences and comparing FP patients with prisoners from the same country, year, and, when possible, the same age span. Even with such adjustments, FP patients still had lower rates of reoffending, including violent recidivism. However, only a few individual comparative studies have compared FP patients with otherwise similar, non-FP offenders within the same legislation and judicial practice. Also, few comparative studies accounted for differential prevalence of confounders in FP and control offender samples: birth year, previous violence, educational and marital status, length of psychiatric hospitalization, and imprisonment (reflecting time at risk for reoffending).

Along with long-term violent reoffending risk estimates in FP patients, information on individual risk factors would help risk assessment and management. Prior research mostly focused on sociodemographic and criminological factors: age, gender, and prior violent offences (27–29). While most individuals with psychiatric disorders do not engage in violence, the risk of committing violence is higher for individuals with a mental disorder than for those without (30). Specifically, links have been established between violent offending and schizophrenia (31, 32), bipolar disorder (33), and psychosis (34) with risks mostly confined to patients with emotional dysregulation, paranoid beliefs, or substance abuse comorbidity (31–33, 35–39). Whether psychiatric disorders are risk factors for reoffending in convicted offenders’ needs, however, further examination (12, 40).

We followed a large, nationally representative Danish cohort of violent and treated FP patients; FP examinees sentenced to FP out- or inpatient treatment at medium secure units (FPE+T). We examined absolute and relative risks of long-term (on average 18+ years at risk) overall, severe, and recurrent violent reoffending compared to 392 individually matched non-examinee non-psychiatric violent general offenders (GEN) and 670 violent non-treated FPE examinees sentenced to ordinary sanctions (FPE-T), respectively. Risk factors for recidivism among FP examinees were identified, and we matched or controlled statistically for unbalanced distributions of possible confounders across cohorts.

## Materials and Methods

### Study Population

Violent offenders referred to court-ordered, pre-trial FP examinations in Copenhagen and Aarhus, Denmark, between 1980 and 1992 were either sentenced to FP treatment (FPE+T; n = 392) or regular non-treatment sanctions (FPE-T; n = 670). We compared FP patients (FPE+T) to matched violent general offender (GEN) control subjects selected from population-based registers run by Statistics Denmark (see matching procedure below).

#### Forensic Psychiatric Examinees

In Denmark, FP evaluations (FPE) are requested to inform court sentencing. FPEs are conducted with selected cases, for example, when a suspected offender has a history of mental illness or shows current psychiatric symptoms or offence-related behaviors that may be signs of severe mental illness. Other determinants include young age (from age 15, the age of criminal responsibility in Denmark) or being elderly (60+ years) as well as indications of a high risk of severe violent reoffending where an indeterminate sanction may be mandatory by law to prevent reoffending (41). The multidisciplinary evaluation leads to a report documenting the mental state of the accused individual and a statement on whether FP care (outpatient or inpatient in moderate or high-security wards, respectively) is recommended. The court independently decides whether to follow the FP team recommendation or not.

FPE examinee data were consecutively collected for a historical population-based cohort of all subjects admitted January 1, 1978, to December 31, 1992, for court-ordered, mandatory pre-trial FP evaluation in Denmark’s two largest cities, at the Department of Forensic Psychiatry, Aarhus, or the Forensic Psychiatry Clinic, Ministry of Justice, Copenhagen, Denmark (N = 2194). Approximately 85% of all FPEs in Denmark during the inclusion period were conducted at these two settings. We included offenders convicted of the violent non-sexual or sexual *index* crime motivating the FP examination and sentenced to either psychiatric treatment (in- or outpatient) or an ordinary sanction, who were possible to be identified in Statistics Denmark registers and did not have an ICD diagnosis of mental retardation (generally IQ < 70). If examined more than once, we selected the first FPE. Importantly, death and emigration were not exclusion criteria; subjects provided follow-up data until death or emigration (when they were censored). Since the Danish Crime Register did not become a research register at Statistics Denmark until 1980, individuals convicted in 1978–1979 were excluded (n = 370). Another 762 (42%) FPE subjects did not meet the inclusion criteria; 128 were not identified in registers (e.g., total population and Danish Psychiatric Central Research Registers), 219 did not have a violent offender control because of narrow matching criteria, 21 were acquitted or sentenced to indeterminate detention or placement at a maximum secure facility, 66 had a diagnosis of mental retardation, and 328 were convicted of a non-violent index offence. The remaining 1,062 violent FPE examinees were divided into two cohorts according to index sentence/sanction: *FP care* (n = 392; treated FPE examinees, FPE+T: 20% were sentenced to outpatient psychiatric care, 43% to inpatient psychiatric care, and 10% to placement in a FP hospital; 24% unknown) or an *ordinary, non-FP sanction* (n = 670; non-treated FPE examinees, FPE-T; 86% were imprisoned and 14% received non-custodial sentences).

#### Matched Violent General Offender Controls

Violent general offender controls (GEN) were individually matched (1:1) to FPE+T individuals on sex, birth year ( ± 3 years), specific violent offence type according to criminal law paragraphs (e.g., if an FPE+T patient was convicted of manslaughter or a sexual offence against a minor; his matched control was indicted or convicted accordingly), and index conviction date (≤2 years within the FPE+T subject’s sentence date) and had never been diagnosed with a psychiatric disorder before or after the violent index offence. We matched on the first four variables according to nationwide register data at Statistics Denmark, while information on the fifth came from the Danish Psychiatric Central Research Register (42). The index sentence for GENs was imprisonment (69%) or non-custodial sentences (31%). Among controls, 69% were imprisoned, and 31% received non-custodial sentences; one control was acquitted.

### Procedures

For all FPE examinees, we obtained baseline offender data from FPE reports and national registers. Six trained raters (SB, JL, and four master-level psychology graduate students trained by SB) extracted sociodemographic information, psychiatric diagnoses, and type and date of index sanction.

Principal ICD-8 psychiatric diagnostic codes were extracted from FPEs according to the International Classification of Diseases (ICD), 8th revision (43) used in Denmark 1966–1994, followed by ICD-10 from 1995 (44). ICD-9 was never implemented in Denmark. Since the WHO did not publish algorithms for translation between ICD-8 and ICD-10, ICD-8 diagnostic codes for Copenhagen and Aarhus FPE subjects were converted to corresponding ICD-10 entities by two senior general psychiatrists, the editor of the psychiatric section of the Danish version of ICD-10 and JL, respectively. We differentiated between major mental disorder categories: *schizophrenia spectrum and other psychotic disorders* (ICD-10: F20–29); *bipolar, depressive, and related disorders* (ICD-10: F30–39); *personality disorders* (ICD-10: F60–61); and *other disorders* (all remaining psychiatric disorders in ICD-10). Complementary diagnostic data for *any substance use disorder* (SUD) were obtained from the Psychiatric Central Research Registers (PCRR) (42) and diagnosed according to ICD-8 (code 303 [alcoholism] and 304 [drug dependency]) or ICD-10 (all ICD-10 F1 disorders except uncomplicated use of a substance [F1x.10], caffeine [F17], or nicotine [F15]). We had no information on psychiatric morbidity including substance abuse in matched violent general offender controls (GEN) because of the “no psychiatric diagnosis” inclusion criterion. Further, data on the specific psychological and pharmacological treatment of major mental and personality disorders in FP examinees and controls were unavailable, both before and after discharge from FP care or release from prison, respectively. Complete treatment data were neither available from registers nor ethically or logistically possible to extract reliably from patient files.

For all subjects, information on most risk factors (sociodemographic, criminal history, and substance use disorder), time-dependent covariates during follow-up (psychiatric hospitalizations, imprisonment, emigration, and death), and outcome measures (criminal reoffending) was obtained from population-based registers. All Danish residents have a unique identification number that was used to link baseline data with high-quality, longitudinal population registers (Statistics Denmark): The National Patient Register, The Crime Register, causes of death, total population, and Psychiatric Central Research Registers (PCRR) (42). The PCRR holds information on all inpatient assessment and treatment, and high validity has been reported for specific PCRR psychiatric diagnoses, including schizophrenia, single episode depression, dementia, and autism (45).

Together with the Swedish Crime Register (31), the Danish Crime Register is one of the most comprehensive and accurate nationwide criminal registries in the Western world (46) and accurately reflects officially resolved criminality by covering all sentences in lower court regardless of type (custodial, noncustodial, FP, etc.) for individuals from 15 years (age of criminal responsibility). Also, *consumption of offences* (that more severe offences can “consume” less severe, so the latter will not be tried and receive a sentence and, hence, not be visible in crime statistics) and *plea bargaining* are seldom practiced in the Danish legal system (47).

### Outcome Measures

Data on all criminal convictions from January 1, 1980 (the earliest date such data were accessible at Statistics Denmark), to December 31, 2011, were retrieved from the Danish Crime Register for the two FPE examinee cohorts and matched violent offender controls. We extracted information on any violent reoffending, offence severity, and frequency of violent sentences. We followed previous studies (31, 48) and applied a broad definition of *any violence* (i.e., any non-sexual [homicide, assault, robbery, arson, illegal threats, and intimidation] or sexual [contact or non-contact, including child sexual exploitation material use/child pornography] offences). Attempted and aggravated offences were counted when applicable. We also differed between *severe violence* (homicide, serious/aggravated assault, robbery, rape, sexual coercion, or child molestation) and *recurrent violence* (defined as 3+ separate court sentences for any violence).

We counted all reconvictions during follow-up, independently of the sentence: ordinary sanctions (e.g., fines, probation, conditional/unconditional imprisonment), preventive measures (e.g., placement or treatment orders, indeterminate detention), and withdrawal of charge. The latter is included since the accused is viewed guilty as charged, although the legal proceedings are completed without trial. Withdrawal of charges is rarely applied, typically for minor offences, for mentally ill offenders already sentenced to treatment at a psychiatric hospital, young offenders, or those already serving time in correction settings (49).

For FPE examinees, reoffending was operationalized as offences committed after the day of FPE completion and for controls as offences committed from the day of the index sentence. First reoffence was the first day of the commission of a violent crime after index. If the date of committing violence was not applicable, date of charge or conviction was applied. We followed subjects until end of follow-up (December 31, 2010), date of death, or emigration, whichever occurred first. Since information on the date of last conditional discharge for treated FP examinees (FPE+T) was not accessible, we calculated time at risk during follow-up as time alive in Denmark not being hospitalized at a psychiatric hospital or imprisoned. Since information on the exact date of release from prison was unavailable for imprisoned offenders (FPE-T and GEN), imprisonment periods were calculated as 2/3 of court-determined prison sentence length; prisoners in Denmark are regularly paroled after serving 2/3 of their sentence.

During follow-up, 43% of FP patients (FPE-T) were censored (41% died, 2% emigrated) *vs*. 27% of GEN (22% died, 5% emigrated) and 40% of FPE-T (37% died, 3% emigrated).

### Statistics

We conducted pairwise comparisons of sample characteristics: first with FP patients (FPE+T) *vs*. matched general offender controls (GEN), and second, with FPE+T *vs*. non-treated FP examinees (FPE-T). Subgroup differences were analyzed with chi-square (categorical data) or t-tests (normally distributed interval or ratio data). Effect sizes are provided as standardized mean differences (Cohen’s d). We estimated treated FP patients’ (FPE+T) reoffending risk relative to controls (GEN) and to FPE examinees receiving ordinary sentences (FPE-T), respectively, using Cox proportional hazard regression. The latter method was also used for survival analyses. In Cox regression models, we adjusted for previous violent sentences and educational and marital status at index offence. Additionally, we either matched (violent offender controls; GEN) or adjusted (untreated FPE examinees; FPE-T) for birth year and sex. Finally, we also used Cox regression to model the long-term predictive ability of potential risk and protective factors on any violent recidivism among all FPE examinees. The latter analysis did not include comparisons with matched violent general offender controls (GEN) since several factors (e.g., substance abuse) were unavailable for them. Across analyses, we compared treated FP patients (FPE+T) to either of the two control groups (i.e., FPE+T *vs*. GEN and FPE+T *vs*. FPE-T).

An independent government agency (Statistics Denmark) merged and pseudonymized the data, and we conducted statistical analyses with SAS version 9.4 (SAS Institute Inc., Cary, NC, USA). The Regional Research Ethics Committee in Aarhus County, Denmark, determined that the study did not need formal ethical approval. We also registered the study with the Danish Data Protection Agency and Region of central Jutland (2002-41-2073, 2007-58-0010, 2011-41-7058, and 1-16-02-530-18).

## Results

The results of pairwise comparisons of sociodemographic, psychiatric, and criminological baseline characteristics of FP patients (FPE+T) *vs*. matched general offender controls (GEN) and FPE+T *vs*. non-treated FP examinees (FPE-T) are presented in **Table 1**. Expectedly, all three cohorts were characterized by single, unemployed males in their thirties with low education and a violent, non-sexual index offence. Overall, we followed subjects for a mean of 20.3 years, without significant differences across cohorts. This corresponded to an overall time-at-risk (i.e., follow-up time when not hospitalized or imprisoned) of 18 to 20 years, with matched general offender controls (GEN) having a slightly longer time-at-risk.

**Table 1:** Characteristics of violent offenders who underwent pre-trial forensic psychiatric evaluation (FPE) in Denmark in 1980–1992 and matched controls.

Characteristic		FPE+T^a^ (N = 392)	GEN^b^ (N = 392)	FPE-T^c^ (N = 670)
**Age, y, mean (SD)**		32.7 (11.5)	32.9 (11.9)^ns^	30.5 (10.1)**
**Male sex, n (%)**		374 (95.4)	374 (95.4)^ns^	635 (94.8)^ns^
**Highest education: 9** **^th^** **grade, n (%)**		240 (61.2)	230 (58.7)**	447 (66.7)***
**Currently employed, n (%)**		86 (21.9)	190 (48.5)***	183 (27.3)*
**Single marital status, n (%)** **^d^**		331 (84.4)	287 (73.2)***	549 (81.9)^ns^
**Principal psychiatric disorder at FPE, n (%)**				
	Schizophrenia spectrum disorder (F2)	225 (57.4)	§	21 (3.1)***
	Bipolar, depressive, and related disorder (F3)	17 (4.3)	§	33 (4.9)
	Personality disorder (F6)	79 (20.2)	§	531 (79.3)
	Other psychiatric diagnosis	68 (17.3)	§	74 (11.0)
	No psychiatric diagnosis	3 (0.8)	392 (100)	11 (1.6)
**Substance abuse comorbidity (F1.1–F1.9), n (%)**		81 (20.7)	§	86 (12.8)***
**Index offence**				
	Any violence^e^, n (%)	317 (80.9)	317 (80.9)^ns^	499 (74.5)*
	Sexual violence^f^, n (%)	75 (19.1)	75 (19.1)^ns^	171 (25.5)*
	Severe non-sexual/sexual violence^g^, n (%)	222 (56.6)	219 (55.9)^ns^	469 (70.0)***
**Prior violence conviction**				
	Any violence, n (%)	75 (19.1)	51 (13.0)**	194 (29.0)***
	No. of violence sentences, mean (SD)	0.28 (0.67)	0.18 (0.53)*	0.50 (0.99)***
	Severe non-sexual/sexual violence, n (%)	40 (10.2)	31 (7.9)^ns^	121 (18.1)***
**Follow-up/time-at-risk**				
	Total follow-up time, y, mean (SD)	20.3 (7.5)	20.5 (7.1) ^ns^	20.6 (8.1)^ns^
	Time in prison, months, mean (SD)^h^	3.1 (14.2)	12.0 (22.5)***	31.6 (33.1)***
	Time in psychiatric hospital, months, mean (SD)	25.6 (40.7)	0 (0)***	3.6 (13.5)***
	Time-at-risk, y, mean (SD)^i^	18.0 (7.6)	19.5 (7.4)***	17.8 (8.3)^ns^

a FPE+T, FPE examinees sentenced to FP treatment.

b GEN, matched violent general offender control subjects.

c FPE-T, FPE examinees sentenced to regular, non-FP treatment, sanctions.

d Single denotes divorced, widowed, or never married.

e Refers to any attempted or completed sexual (any sexual contact or non-contact offence) or non-sexual (homicide, violent assault, robbery, arson, unlawful threats, or offences against personal liberty) violent offence.

f Refers to any attempted or completed sexual offence (contact or non-contact).

g Denotes any attempted or completed homicide, aggravated assault, robbery, rape, sexual coercion, and child molestation.

h Calculated as 2/3 of the total prison sentence length since prisoners in Denmark are generally paroled after having served this proportion of the full sentence.

i Refers to time at risk during follow-up (excluding time imprisoned or hospitalized within Danish psychiatric hospital-based services).

§, is zero by definition of the control group.

ns, non-significant, p ≥ 0.05; *p < 0.05; **p < 0.01; ***p < 0.001.

FP patients (FPE+T) were characterized by more pre-index violence (any violence and number of violent convictions) and less protective factors (higher educational level, current employment, having a partner) compared to GENs. As opposed to GEN, all FP patients were diagnosed with at least one psychiatric disorder; a total of 21% also had a comorbid substance use disorder (SUD) diagnosis.

Untreated FPE examinees (FPE-T) were, on the contrary, characterized by far more known risk factors (significantly younger age, lower educational level, personality disorder [PD] diagnosis, higher rates of any violence and severe violence, and number of violent sentences) as compared to treated FP patients (FPE+T). Yet, a significantly larger proportion of the latter had a SUD diagnosis than FPE-T.

Absolute and relative rates [adjusted hazard (any and severe violence) or odds ratios (3+ violent sentences)] for facets of violent reoffending are reported in **Table 2**. Some bivariate between-group differences in risk disappeared upon adjustment for previous violence, education and marital status, birth year, and sex. Violent recidivism rates were higher for FPE+T as compared to GENs for any violence (43% *vs*. 29%; aHR = 1.5; 95% CI, 1.1–1.9), severe (21% *vs*. 14%; aHR = 1.3; 95% CI, 0.9–1.9), and recurrent violence (16% *vs*. 6%; aOR = 2.5; 95% CI, 1.5–4.4) but did not reach statistical significance (p < 0.05) for severe violence. Reoffending risk was lower for FPE+T patients than FPE-T examinees for any violence (43% *vs*. 51%; aHR = 0.9; 95% CI, 0.7–1.0), severe violence (21% *vs*. 34%; aHR = 0.7; 95% CI, 0.5–0.9), and recurrent violence (16% *vs*. 22%; aOR = 0.7; 95% CI, 0.5–1.0) than for non-treated FPE examinees, albeit running short of formal statistical significance for any violence and recurrent violence. To assess the robustness of Cox regression findings, we also conducted corresponding multivariable logistic regression analyses, finding essentially comparable results as with the Cox models (data not shown).

**Table 2:** Facets of violent recidivism until 2010 in violent offenders who underwent pre-trial forensic psychiatric evaluation (FPE) in Denmark 1980–1992 and matched controls.

Reoffending facet	Reoffending rate (%, n)			Relative reoffending risk in FPE+T compared to:	
	FPE+^a^ (N = 392)	GEN^b^ (N = 392)	FPE-T^c^ (N = 670)	GEN^g^ Adjusted [a] HR/OR (95% CI)	FPE-T^h^ Adjusted [a] HR/OR (95% CI)
Any violence^d^	43% (168)	29% (114)^§^***	51% (344)^§^**	aHR = 1.5 (1.1–1.9)**	aHR = 0.8 (0.6–1.1)^ns^
Severe violence^e^	21% (84)	14% (54)^§^**	34% (225)^§^***	aHR = 1.3 (0.9–1.9)^ns^	aHR = 0.6 (0.4–0.8)**
Recurrent violence^f^	16% (63)	6% (24)^§^***	22% (147)^§ns^	aOR = 2.5 (1.5–4.4)***	aOR = 0.7 (0.5–1.0)^ns^

ns, non-significant, p ≥ 0.05; *p < 0.05; **p < 0.01; ***p < 0.001.

a FPE+T, FPE examinees sentenced to FP treatment.

b GEN, matched violent general offender control subjects.

c FPE-T, FPE examinees sentenced to regular, non-FP treatment, sanctions.

d Refers to any attempted or completed sexual (any sexual contact or non-contact offence) or non-sexual (homicide, violent assault, robbery, arson, unlawful threats, or offences against personal liberty) violent offence.

e Denotes homicide, aggravated assault, rape, sexual coercion, child molestation, and robbery.

f Defined as at least three separate sentences for any violence during follow-up.

^§^Pairwise chi-square comparison of violent reoffending rates: FP patients (FPE+T) vs. matched general offender controls (GEN) and non-treated FP examinees (FPE-T), respectively. aHR/aORs > 1 indicate higher reoffending among FPE+T patients than GEN or FPE-T subjects, respectively, while aHR/aORs < 1 indicate lower reoffending rates in FPE+T patients.

aHR, adjusted hazard ratio; obtained with Cox proportional hazard regression modeling accounting for varying time at risk and other covariates mentioned under (g). aOR, adjusted odds ratio; obtained with logistic regression and adjusted for covariates mentioned under (h). CI, confidence interval.

g We also adjusted analyses for any previous conviction of violence, highest education, and marital status at FPE.

h We did not match these subjects with FPE+T patients. However, comparisons were similarly adjusted for birth year, sex, any previous violent conviction, highest education, and marital status at FPE.

**Figure 1:**
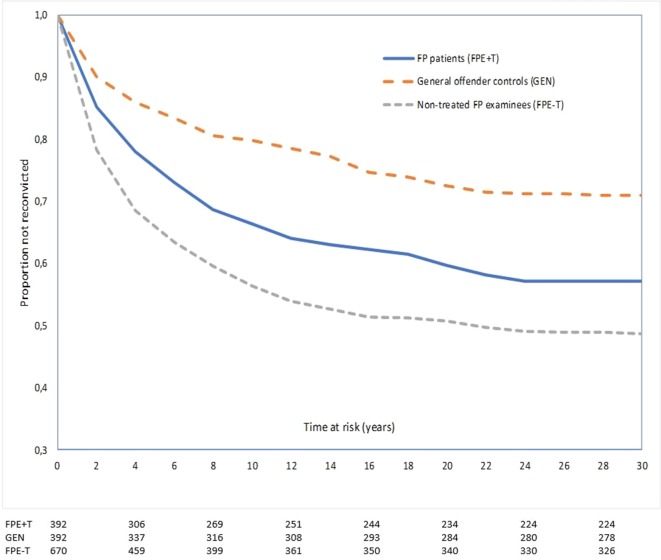
Proportion not reconvicted for any violent reoffending as a function of time at risk among violent offenders who underwent pre-trial forensic psychiatric evaluation (FPE) in Denmark 1980–1992. We compared 392 FPE examinees consequently treated (+T) in forensic psychiatry (FPE+T), 392 matched violent general offender controls (GEN), and 670 FPE examinees with ordinary sanctions (e.g., prison) treated as usual outside forensic psychiatry (FPE-T). Numbers below graphs represent the remaining number of non-censored subjects that had not recidivated at the beginning of each 4-year interval indicated on the X-axis.

Kaplan–Meier survival curves suggested similar violent reoffending trajectories for the first 2 years across all three groups while reconvictions were quite unlikely beyond about 20 years, despite substantial numbers of individuals still at risk (**Figure 1**).

A Cox regression model identified weak to moderately strong independent effects of male sex, previous violent crime, multiple admissions to psychiatric hospital (5+) during follow-up, SUD, younger age, and personality disorder as main diagnosis (as compared to schizophrenia spectrum disorders and “other psychiatric disorders”) on violent reoffending among FPE examinees, independently of their medico-legal status (involving a conviction to FP care or an ordinary sentence) (**Table 3**).

**Table 3:** Multivariable Cox regression analysis of risk factors for any violent reconviction among 1,062 violent offenders subjected to pre-trial forensic psychiatric evaluation (FPE) in Denmark during 1980–1992, followed for 18–31 years.^a^,^b^

Risk factor	Adjusted hazard ratio	95% CI
Male sex *vs*. not	**2.08**	1.3–3.5**
Age (y)^c^	**0.95**	0.9–1.0***
Married *vs*. not	0.79	0.6–1.0^ns^
Unemployed *vs*. not	1.21	1.0–1.5^ns^
Pre-index violent conviction *vs*. not	**1.95**	1.6–2.3***
Personality disorder (F6)	Reference	
Schizophrenia spectrum disorders (F2) *vs*. F6	**0.49**	0.4–0.7***
Bipolar, depressive, and related disorders (F3) *vs*. F6	1.17	0.8–1.7^ns^
Other psychiatric diagnosis *vs*. F6	**0.76**	0.6–1.0*
No psychiatric diagnosis *vs*. F6	0.99	0.4–2.2^ns^
(Severe) substance use disorder (SUD) (F1.1–F1.9) *vs*. not	**1.29**	1.0–1.6*
No admissions to psychiatric hospital after index	Reference	
1–4 admissions to psychiatric hospital after index *vs*. no admission	1.24	1.0–1.6^ns^
5+ admissions to psychiatric hospital after index *vs*. no admission	**1.95**	1.5–2.5***

We obtained adjusted hazard ratios from multivariable Cox proportional hazards regression modeling accounting for varying time at risk and all other predictors included in the model. Bolded figures are significant at p < 0.05. CI, confidence interval.

ns, non-significant, p ≥ 0.05; *p < 0.05; **p < 0.01; ***p < 0.001.

a Following forensic psychiatric evaluation: examinees received either psychiatric care or ordinary (non-psychiatric) sanctions.

b N = 1,055, 7 subjects had data missing on one or more risk factor and were consequently excluded from the Cox regression.

c Interpretation: since age is a continuous variable, the hazard for recidivism decreases by 0.95 per one-year increase in age. This equals a (1–0.95^10^)/100 = 40% lower risk in individuals 10 years older than others at baseline, while accounting also for all other tested risk factors in the model.

## Discussion

This controlled, nationwide, long-term follow-up study addressed rates and facets of violent reoffending in violent FPE examinees treated in forensic psychiatry (FPE+T) relative to two comparison cohorts of violent offenders. Violent offenders were consecutively referred for court-ordered pre-trial FPE in Aarhus and Copenhagen, Denmark, between 1980 and 1992 (FPE examinees) or drawn from nationwide longitudinal registers [violent general offender controls (GEN) individually matched to FPE+T]. During an 18+ year follow-up, the absolute risk of any violent reoffending was 43% for treated FP patients (FPE+T), 29% for GENs, and 51% for non-treated FPE examinees (FPE-T). We found a similar trend for reoffending facets severe and recurrent violent reoffending; GENs had the lowest reoffending risk followed by FP patients and non-treated FPE examinees.

Base rates of violent reoffending in FP patients were similar to those reported in prior longer-term studies of FP patients, 46% (14) and 40% (12). While length of follow-up and base rate in Lund et al. (14) were comparable to ours, Pedersen and colleagues (16) reported a high 40% violent reconviction rate during a substantially shorter 5.6-year follow-up of 107 FP patients discharged from a medium secure FP unit in Denmark in 2001–2002. The base rate in Pedersen et al. may reflect that FP patients followed more recently may experience increased reporting of psychiatric patients’ violent episodes against staff leading to higher observed rates of violent re-offending in FP patients (49). Alternatively, the base rate suggests faster reoffending and then little additional recidivism after a longer follow-up, or the sample consisted of a more recidivism-prone group of treated FP patients.

Prior comparative studies typically reported *lower* violent reoffending rates in FP patients than among non-FP general offenders, such as prisoners with similar ages, lengths of stay, and offence types (12, 15, 50). In contrast, a smaller long-term Swedish study reported higher violent reoffending rates in FP patients than what we have found, although not significantly higher than that for offenders with other sanctions (14). Different relative risks across studies of FP patients *vs*. controls might be related to variations in sample composition. As discussed by Fazel and colleagues (12), many FP patients exhibit factors linked to *lower* reoffending risk. These include later onset of offending, fewer prior convictions, more severe index offences, shorter time-at-risk to reoffend because of extended hospitalization periods, and access to psychiatric treatment. Further, primary drivers of (re)offending may be acute symptoms of severe mental illness rather than criminogenic aspects characterizing general offenders, factors rarely controlled for in research. Further, the source of admission (court *vs*. community) was not reported in most studies in the Fazel et al. review (12). We followed offenders sentenced to psychiatric care by the court. Some research—for example, UK studies of patients discharged from medium secure settings may also include complex general psychiatric patients that were placed in medium secure settings, without technically being “true” FP patients.

We tried to overcome some of the limitations of prior studies. After matching and adjusting for several known confounders, FP patients were found to have significantly higher risks of any violence and recurrent violence than non-FP, general offender controls (GEN). Time at risk was quite similar for the cohorts; thus, its variability could not explain FP patients’ increased risk. GENs, however, had longer education, were more often employed and had an intimate partner than both treated FP patients and non-treated FPE examinees. Hence, despite controlling for covariates, our matched general offender controls remained more high functioning than FP patients. And, possibly, due to the matching on index offence, they were even more well-functioning than violent non-FP general offenders. That is, the index offence may have been representative for FP patients but less so for general offenders. Contrarily, non-treated FPE examinees were characterized by factors typically found among general offenders: younger age, poorer education, PD diagnoses, and more extensive prior violence including severe and repeated violence. Hence, our non-treated FPE examinees may have been more similar general offenders included in prior comparative studies, although with more violent reoffending than commonly reported (51, 52). The elevated recidivism rates suggest that FPE referral partly and inadvertently selected high-risk offenders with high sensitivity. Expectedly, several of these risk characteristics pertain to the recidivism risk factor domains (i.e., the Central Eight) summarized by Andrews and Bonta (53): history of antisocial behavior, antisocial personality pattern, substance abuse, poor family/marital relationships, and school/work problems. However, we were unable to systematically collect data on antisocial cognitions and associates, two of the four most predictive domains of criminal recidivism. The risk principle, one of three widely accepted principles for evidence-based, recidivism-reducing services (53) specifically prescribe that more extensive rehabilitative efforts should be directed toward offenders with more risk factors. Our findings suggest a need for continua of service for FPE examinees independently of their medico-legal status, that is, sentenced to inpatient FP treatment or not. Systematic reviews suggest high rates of psychiatric disorders also among prisoners (54), including substance misuse (55).

It may be tempting to interpret lower reoffending risk in treated *vs*. untreated FP examinees as an indication of a positive effect of FP care. However, causal conclusions should be avoided, first, because the weak to moderate differences in violent recidivism among treated FP patients as compared to non-treated FPE examinees became nonsignificant after statistical adjustment of well-known confounders (pre-index violence, education, and marital status). Second, FP examinees with 5+ admissions to a psychiatric hospital after the index conviction had almost twice the risk (adjusted HR = 1.95; 95% CI: 1.5–2.5) of violent recidivism compared to those with no hospital admissions. Similarly, this should not be interpreted to indicate that treatment *increased* violence risk. This finding may rather reflect additional unmeasured risk in those admitted more often, such as disorder severity and negative symptoms with or without concomitant medication non-adherence or substance abuse—both increasing the need for psychiatric care. To put differently, the distribution of subjects to FP treatment *vs*. ordinary non-FP sanctions is not randomized but purposefully sorted according to legislation and judicial practice. Hence, residual confounding cannot be excluded; that is, treated and non-treated FPE examinees likely differ in ways that were not possible to control for.

We also determined risk factors for violent re-offending among all FPE examinees, independently of a subsequent sentence to FP treatment or not. The analysis found male sex, younger age, previous violent crime, personality disorder (as compared to schizophrenia spectrum disorders and other psychiatric diagnoses), substance use disorder, and multiple psychiatric admissions to independently predict violent recidivism. These are all established criminogenic factors (14, 28, 29, 31, 35, 54, 56–58).

So far, there is uncertainty over the causes and the remaining heterogeneity found for the association between major mental disorder and violence. Variations across studies in age, SES, and comorbid substance use disorder or personality disorder might be the most plausible explanations of the mixed results (34). Studies aimed at identifying causal and potentially changeable risk factors specifically for FP patients are indeed needed.

### Clinical Implications

First, although the ethics of violence risk assessment remains debated (59), substantial long-term violent reoffending among FP patients may motivate continued efforts to improve assessment and provision of evidence-based management of violence risk. Although structured risk assessments show higher reliability and predictive validity than unstructured clinical judgments, low risk-individuals are still more accurately identified than high-risk offenders (60). Prevention of reoffending requires addressing also dynamic risk factors and criminogenic needs, potentially amenable to intervention. So-called third and fourth generation risk assessment tools [e.g., Historical Clinical Risk-20 [HCR-20] V3; (61) Level of Service/Case Management Inventory (LS/CMI) (62)] aim at focusing a broader range of risk factors and integrate monitoring and intervention. However, such assessments are time-consuming and subsequently difficult to employ on a large scale in everyday clinical practice. Future research should examine how much added value to risk prevention more comprehensive risk assessments do indeed provide (63).

### Limitations

First, historical data may be measured or documented inconsistently, which affects internal validity. Then again, we extracted baseline information from structured FPE protocols as documented 1980–1992 from accurate nationwide registers and followed the three cohorts until 2010. Second, we selected matched violent general offender controls from nationwide registers. Psychiatric disorders are common in prisoners worldwide (64), and controls might have suffered from psychiatric ill-health undetected in psychiatric registers. Third, detailed treatment data for FP patients and controls were neither available from registers nor possible to extract logistically and reliably from clinical records. This was the case for periods both before and after discharge from FP care and release from prison. However, throughout the long observation period, aftercare for discharged FP patients (and FP examinees who received non-FP sanctions) was generally provided by general adult psychiatry in the subject’s respective district of residence. Regarding prisoners, we are not aware of *systematic* corrections-based psychological treatments during the study period; thereby not excluding likely clinically motivated treatment. Fourth, data specifically on violent acts toward staff, and other patients during inpatient psychiatric care were not detectable unless reported to the police and registered in the Crime Register. Fifth, since data on criminal convictions were unavailable before 1980, the pre-index violence variable underestimated actual prevalence. It is, however, reasonable to assume that underestimation may be similar in the three cohorts and so should only affect estimate precision, not effect sizes. Sixth and finally, our study did not encompass also the large group of likely recidivism-prone offenders not referred for FPE, but who do suffer from mental disorders such as antisocial personality disorder and SUDs.

## Conclusions

Our results suggest persisting violent reoffending during extended periods in FPE examinees; in treated followed by non-treated FPE examinees. FPE examinees also had a disproportionately high-violent reoffending risk and high risks of severe and recurrent violence. Violent recidivism was optimally predicted by established criminogenic factors rather than having a severe mental disorder. Our findings suggest a need for continua of service for FPE examinees, independently of whether they are treated as FP inpatients or not. Management of violent reoffending risk in FP patients may need a longer-term perspective, as recidivistic violence occurred up until ca 20 years of follow-up. To guide the complex task to care clinically for FP patients, future outcome studies may benefit from finding and reducing the impact of causal, changeable risk factors to various facets of violent reoffending.

## Data Availability Statement

Requests for data can be made to Dr. Susanne Bengtson Pedersen. Such requests would be evaluated on a case by case basis as the dataset is being used for other studies. According to strict policies employed by data provider Statistics Denmark, requesting persons also have to be individually approved by this body.

## Ethics Statement

Ethical review and approval was not required for the study on human participants in accordance with the local legislation and institutional requirements. Written informed consent for participation was not required for this study in accordance with the national legislation and the institutional requirements.

## Author Contributions

SB and JL designed the study and collected the data. SB took the lead in writing the manuscript, although in close collaboration with and under supervision of NL. MI performed the statistical calculations. All authors provided critical feedback and helped shape design, analyses, interpretation and manuscript.

## Funding

We followed a cohort within the Danish PAK-study (Study of Mentally Disordered Offenders) which received generous financial support from The Danish Health Foundation, the National Institute for Health and Medical Research, the Foundation for Advancing Psychiatric Research at the Psychiatric Hospital, Risskov, Aarhus University Research Fund, The Ivan Nielsen Fund, The Færgeman Grant, the Mental Health Promotion Fund and the Master butcher Max Wørzner and his wife Inger Wørzner´s Commemorative Grant for Promoting Mental Disease Research. None of the funders were involved in study design, data analyses, conclusions, or the decision to publish.

## Conflict of Interest

The authors declare that they conducted the research in the absence of any commercial or financial relationship that could be construed as a potential conflict of interest.

## References

[1] Kramp P Gabrielsen G The organization of the psychiatric service and criminality committed by the mentally ill. Eur Psychiatry (2009) 24:401–11. 10.1016/j.eurpsy.2009.07.007 19720504

[2] Wolf A Fanshawe TR Sariaslan A Cornish R Larsson H Fazel S Prediction of violent crime on discharge from secure psychiatric hospitals: a clinical prediction rule (FoVOx). Eur Psychiatry (2018) 47:88–93. 10.1016/j.eurpsy.2017.07.011 29161680 PMC5797975

[3] Chow WS Priebe S How has the extent of institutional mental healthcare changed in Western Europe? Analysis of data since 1990. BMJ Open (2016) 29(6(4)):e010188. 10.1136/bmjopen-2015-010188 PMC4854016 27130161

[4] Priebe S Badesconyi A Fioritti A Hansson L Kilian R Torres-Gonzales F Reinstitutionalisation in mental health care: comparison of data on service provision from six European countries. BMJ (2005) 303:123–6. 10.1136/bmj.38296.611215.AE PMC544427 15567803

[5] Völlm BA Edworthy R Huband N Talbot E Majid S Holley J Characteristics and pathways of long-stay patients in high and medium secure settings in England. Front Psychiatry (2018) 9:140. 10.3389/fpsyt.2018.00140 29713294 PMC5911489

[6] Møllerhøj J Stølan LO Brandt-Christensen M A thorn in the flesh? Forensic inpatients in general psychiatry. Perspect Psychiatr Care (2016) 52(1):32–9. 10.1111/ppc.12099 25624050

[7] Douglas KS Ogloff JRP Multiple facets of fisk for violence: the impact of judgmental specificity on structured decisions about violence risk. Int J Forensic Ment Health (2003) 2:19–34. 10.1080/14999013.2003.10471176

[8] Kip H Bouman YHA Kelders SM van Gemert-Pijnen LJEWC eHealth in treatment of offenders in forensic mental health: a review of the current state. Front Psychiatry (2018) 9:42. 10.3389/fpsyt.2018.00042 29515468 PMC5826338

[9] Rutherford M Duggan S Forensic mental health services: facts and figures on current provision. Br J Forensic Pract (2008) 1(10(4)):4–10. 10.1108/14636646200800020

[10] O’Neill C Heffernan P Goggins R Corcoran C Linehan S Duffy D Long-stay forensic psychiatric inpatients in the Republic of Ireland: aggregated needs assessment. Ir J Psychol Med (2003) 20:119–25. 10.1017/S0790966700007916 30308720

[11] Fazel S Fimińska Z Cocks C Coid J Patient outcomes following discharge from secure psychiatric hospitals: systematic review and meta-analysis. Br J Psychiatry (2016) 208(1):17–25. 10.1192/bjp.bp.114.149997 26729842 PMC4698562

[12] Fazel S Wolf A Fimińska Z Larsson H Mortality, rehospitalisation and violent crime in forensic psychiatric patients discharged from hospital: rates and risk factors. PloS One (2016) 11:e0155906. 10.1371/journal.pone.0155906 27196309 PMC4873227

[13] Tabita B de Santi MG Kjellin L Criminal recidivism and mortality among patients discharged from a forensic medium secure hospital. Nord J Psychiatry (2012) 66:283–9. 10.3109/08039488.2011.644578 22212020

[14] Lund C Hofvander B Forsman A Anckarsäter H Nilsson T Violent criminal recidivism in mentally disordered offenders: follow-up study of 13–20 years through different sanctions. Int J Law Psychiatry (2013) 36:250–7. 10.1016/j.ijlp.2013.04.015 23672945

[15] Nilsson T Wallinius M Gustavson C Anckarsäter H Kerekes N Violent recidivism: a long-time follow-up study of mentally disordered offenders. PLoS One (2011) 6(10):e25768. 10.1371/journal.pone.0025768 22022445 PMC3191156

[16] Pedersen L Rasmussen K Elsass P Risk assessment: the value of structured professional judgments. Int J Forensic Ment Health (2010) 9(2):74–81. 10.1080/14999013.2010.499556

[17] Skipworth J Brinded P Chaplow D Frampton C Insanity acquittee outcomes in New Zealand. Aust N Z J Psychiatry (2006) 40:1003–9. 10.1080/j.1440-1614.2006.01924.x 17054569

[18] Centre for Mental Health Pathways to unlocking secure mental health care. UK, London: Centre for Mental Health (2011).

[19] Webster CD Douglas KS Eaves D Hart SD HCR-20: Assessing risk for violence, version 2. Burnaby, British Colombia: Mental Health, Law and Policy Institute, Simon Fraser University (1997).

[20] Monahan J The clinical prediction of violent behavior. Washington, DC: Government Printing (1981). 10.1037/e664392007-001

[21] Kahneman D Tversky A On the psychology of prediction. Psychol Rev (1973) 80(4):237–51. 10.1037/h0034747

[22] Coid J Hickey N Kahtan N Zhang T Yang M Patients discharged from medium secure forensic psychiatry services: reconvictions and risk factors. Br J Psychiatry (2007) 190:223–9. 10.1192/bjp.bp.105.018788 17329742

[23] Sjöstedt G Grann M Risk assessment: what is being predicted by actuarial prediction instruments? Int J Forensic Ment Health (2002) 1:179–83. 10.1080/14999013.2002.10471172

[24] Långström N Long-term follow-up of criminal recidivism in young sex offenders: temporal patterns and risk factors. Psychol Crime Law (2002) 8:41–58. 10.1080/10683160208401808

[25] Bengtson S Långström N Unguided clinical and actuarial assessment of re-offending risk: a direct comparison with sex offenders in Denmark. Sex Abuse J Res Treat (2007) 19:135–53. 10.1007/s11194-007-9044-5 17534713

[26] Fazel S Wolf A A systematic review of criminal recidivism rates worldwide: current difficulties and recommendations for best practice. PLoS One (2015) 10(6):e0130390. 10.1371/journal.pone.0130390 26086423 PMC4472929

[27] Bonta J Blais J Wilson HA A theoretically informed meta-analysis of the risk for general and violent recidivism for mentally disordered offenders. Aggress Violent Behav (2014) 19:278–87. 10.1016/j.avb.2014.04.014

[28] Collins RE The effect of gender on violent and nonviolent recidivism: a meta-analysis. J Crim Justice (2010) 38(4):675–84. 10.1016/j.jcrimjus.2010.04.041

[29] Bonta J Law M Hanson K The prediction of criminal and violent recidivism among mentally disordered offenders: a meta-analysis. Psychol Bull (1998) 123:123–42. 10.1037//0033-2909.123.2.123 9522681

[30] Silver E Understanding the relationship between mental disorder and violence: the need for a criminological perspective. Law Hum Behav (2006) 30:685–706. 10.1007/s10979-006-9018-z 16972182

[31] Fazel S Gulati G Linsell L Geddes JR Grann M Schizophrenia and violence: systematic review and meta-analysis. PLoS Med (2009) 6(8):e1000120. 10.1371/journal.pmed.1000120 19668362 PMC2718581

[32] Fazel S Långström N Hjern A Grann M Lichtenstein P Schizophrenia, substance abuse, and violent crime. JAMA (2009) 301(19):2016–23. 10.1001/jama.2009.675 PMC4905518 19454640

[33] Fazel S Lichtenstein P Grann M Goodwin GM Långström N Bipolar disorder and violent crime: new evidence from population-based longitudinal studies and systematic review. Arch Gen Psychiatry (2010) 67(9):931–8. 10.1001/archgenpsychiatry.2010.97 20819987

[34] Douglas KS Guy LS Hart SD Psychosis as a risk factor for violence to others: a meta-analysis. Psychol Bull (2009) 135(5):679–706. 10.1037/a0016311 19702378

[35] Elbogen EB Johnson SC The intricate link between violence and mental disorder. Arch Gen Psychiatry (2009) 66:152. 10.1001/archgenpsychiatry.2008.537 19188537

[36] Castillo ED Fiftal Alarid L Factors associated with recidivism among offenders with mental illness. Int J Offender Ther Comp Criminol (2011) 55(1):98–117. 10.1177/0306624X09359502 20181775

[37] O’Driscoll C Larney S Indig D Basson J The impact of personality disorders, substance use and other mental illness on re-offending. J Forens Psychiatry Psychol (2012) 23(3):382–91. 10.1080/14789949.2012.686623

[38] Wilson AB Draine J Hadley T Metraux S Evans A Examining the impact of mental illness and substance use on recidivism in a county jail. Int J Law Psychiatry (2011) 34:264–68. 10.1016/j.ijlp.2011.07.004 21839518

[39] Skeem JL Monahan J Mulvey EP Psychopathy, treatment involvement, and subsequent violence among civil psychiatric patients. Law Hum Behav (2002) 26(6):577–603. 10.1023/A:1020993916404 12508696

[40] Chang Z Larsson H Lichtenstein P Fazel S Psychiatric disorders and violent reoffending: a national cohort study of convicted prisoners in Sweden. Lancet Psychiatry (2015) 2:891–900. 10.1016/S2215-0366(15)00234-5 26342957 PMC4629414

[41] Kramp P Denmark. In Salize H. J. Dressing H (eds.). Mentally disordered persons in European prison systems - needs, programmes and outcome (EUROPRIS). Research project, final report. Mannheim, Germany: Pabst Science Publishers (2009).

[42] Munk-Jørgensen P Mortensen PB The Danish Psychiatric Central Register. Dan Med Bull (1997) 44:82–4. 9062767

[43] World Health Organization (WHO) ICD-8. Klassifikation af sygdomme; Udvidet dansk-latinsk udgave af verdenssundhedsorganisationens internationale klassifikation af sygdomme [eng: extended Danish-Latin version of the WHO International Classification of Diseases. 8th revision). Copenhagen, Denmark: Danish National Board of Health (1965).

[44] World Health Organization (WHO) ICD-10: Psykiske lidelser og adfærdsmæssige forstyrrelser. Klassifikation og diagnosekriterier [WHO ICD-10: Mental and Behavioural Disorders. Classification and Diagnostic Criteria]. Copenhagen, Denmark: Munksgaard (1994).

[45] Mors O Perto GP Mortensen PB The Danish Psychiatric Central Research Register. Scand J Public Health (2011) 39(7_suppl):54–7. 10.1177/1403494810395825 21775352

[46] Brennan PA Mednick SA Hodgins S Major mental disorders and criminal violence in a Danish birth cohort. Arch Gen Psychiatry (2000) 57(5):494–500. 10.1001/archpsyc.57.5.494 10807490

[47] Toftegaard Nielsen G Straffesagens gang. 3. udgave. (eng: Criminal proceedings). Copenhagen, Denmark: Christian Ejlers (2004).

[48] Harris G Rice M Quinsey V Violent recidivism of mentally disordered offenders: the development of a statistical prediction instrument. Crim Just Behav (1993) 20(4):315. 10.1177/0093854893020004001

[49] Sundheds- og ældreministeriet (SUM) Kortlægning af retspsykiatrien: mulige årsager til udviklingen i antallet af retspsykiatriske patienter samt viden om indsatser for denne gruppe. (eng: Mapping forensic psychiatry: possible reasons for the increase in the number of forensic psychiatric patients and principles of preventive measures for this group). Copenhagen, Denmark: Ministry of Health (2015).

[50] Fazel S Chang Z Fanshawe T Långström N Lichtenstein P Larsson H Prediction of violent reoffending on release from prison: derivation and external validation of a scalable tool. Lancet Psychiatry (2016) 3(6):535–43. 10.1016/S2215-0366(16)00103-6 PMC4898588 27086134

[51] Durose MR Cooper AD Snyder HN. Recidivism of prisoners released in 30 states in 2005: patterns from 2005 to 2010. Washington, DC: US Department of Justice, Office of Justice Programs, Bureau of Justice Statistics NCJ (2014). p. 244205.

[52] UK Ministry of Justice 2012 compendium of re-offending statistics and analysis. London, UK (2012)

[53] Andrews DA Bonta J The psychology of criminal conduct. 5th ed Cincinnati, OH, USA: Anderson Publishing Co (2010).

[54] Fazel S Baillargeon J The health of prisoners. Lancet (2011) 377(9769):956–65. 10.1016/S0140-6736(10)61053-7 21093904

[55] Fazel S Bains P Doll H Substance abuse and dependence in prisoners: a systematic review. Addiction (2006) 101(2):181–91. 10.1111/j.1360-0443.2006.01316.x 16445547

[56] Tengström A Hodgins S Kullgren G Men with schizophrenia who behave violently: the usefulness of an early-versus late-start offender typology. Schizophr Bull (2001) 27(2):205–18. 10.1093/oxfordjournals.schbul.a006867 11354588

[57] Skeem JL Winter E Kennealy PJ Louden JE Tatar JR Offenders with mental illness have criminogenic needs, too: toward recidivism reduction. Law Hum Behav (2014) 38(3):212–24. 10.1037/lhb0000054 24377913

[58] Monahan J Rethinking risk assessment: the MacArthur study of mental disorder and violence. United Kingdom: Oxford University Press (2001).

[59] McSherry B Managing fear: the law and ethics of preventive detention and risk assessment. New York, NY: Routledge (2014). 10.4324/9780203095652 PMC4226420 25431531

[60] Fazel S Singh JP Doll H Grann M Use of risk assessment instruments to predict violence and antisocial behaviour in 73 samples involving 24 827 people: systematic review and meta-analysis. BMJ (2012) 345:e4692. 10.1136/bmj.e4692 22833604 PMC3404183

[61] Douglas KS Hart SD Webster C Belfrage H HCR-20V3: Assessing risk for violence. Burnaby, Canada: Mental Health, Law, and Policy Institute, Simon Fraser University (2013).

[62] Andrews DA Bonta J Wormith J The Level of Service/Case Management Inventory. Newark, NJ: LexisNexis (2004).

[63] Singh JP Desmarais SL Hurducas C Arbach-Lucioni K Condemarin C Dean K International perspectives on the practical application of violence risk assessment: a global survey of 44 countries. Int J Forensic Ment Health (2014) 13:193–206. 10.1080/14999013.2014.922141

[64] Fazel S Hayes AJ Bartellas K Clerici M Trestman R Mental health of prisoners: prevalence, adverse outcomes, and interventions. Lancet Psychiatry (2016) 3(9):871–81. 10.1016/S2215-0366(16)30142-0 PMC5008459 27426440

